# Patterns of sedentary behavior in overweight and moderately obese users of the Catalan primary-health care system

**DOI:** 10.1371/journal.pone.0190750

**Published:** 2018-01-25

**Authors:** Elena Martínez-Ramos, Angela-Maria Beltran, Carme Martín-Borràs, Lourdes Lasaosa-Medina, Jordi Real, José-Manuel Trujillo, Mercè Solà-Gonfaus, Elisa Puigdomenech, Eva Castillo-Ramos, Anna Puig-Ribera, Maria Giné-Garriga, Noemi Serra-Paya, Beatriz Rodriguez-Roca, Ana Gascón-Catalán, Carlos Martín-Cantera, for the SEDESTACTIV group

**Affiliations:** 1 Primary Healthcare Centre Vilanova 1, Institut Català de la Salut (ICS), Barcelona, Spain; 2 Lifestyles Study Group,RedIAPP, Institut Universitari d'Investigació en Atenció Primària Jordi Gol (IDIAP Jordi Gol), Barcelona, Spain; 3 Department of Physical Activity and Sport Sciences, FPCEE Blanquerna, Universitat Ramon Llull (URL), Barcelona, Spain; 4 Department of Physical Therapy, FCS Blanquerna, URL, Barcelona, Spain; 5 Escuela Superior de Ciencias de la Salud TecnoCampus Mataró-Maresme, Universidad Pompeu Fabra, Barcelona, Spain; 6 Primary Healthcare Centre Passeig Sant Joan, ICS, Barcelona, Spain; 7 Unitat de Suport a la Recerca, Institut Universitari d'Investigació en Atenció Primària Jordi Gol (IDIAP Jordi Gol), Barcelona, Spain; 8 Facultat de Medicina i Ciències de la Salut, Universitat Internacional de Catalunya, Sant Cugat, Spain; 9 Primary Healthcare Centre Cuevas del Almanzora, Almeria, Spain; 10 Primary Healthcare Centre Les Planes, ICS, Barcelona, Spain; 11 Department of Medicine, Universitat Autònoma de Barcelona, Bellaterra, Barcelona, Spain; 12 Primary Healthcare Centre Molí Nou, ICS, Barcelona, Spain; 13 Research Group in Sport and Physical Activity, Health and Social Studies Centre, Universitat de Vic-Universitat Central de Catalunya (UVic-UCC), Vic, Spain; 14 Department of Physiatry and Nursing, Universidad de Zaragoza, Zaragoza, Spain; National Institutes of Biomedical Innovation, Health and Nutrition, JAPAN

## Abstract

**Background and objectives:**

Prolonged sitting time (ST) has negative consequences on health. Changing this behavior is paramount in overweight/obese individuals because they are more sedentary than those with normal weight. The aim of the study was to establish the pattern of sedentary behavior and its relationship to health, socio-demographics, occupation, and education level in Catalan overweight/obese individuals.

**Methods:**

A descriptive study was performed at 25 healthcare centers in Catalonia (Spain) with 464 overweight/moderately obese patients, aged25 to 65 years. Exclusion criteria were chronic diseases which contraindicated physical activity and language barriers. Face-to-face interviews were conducted to collect data on age, gender, educational level, social class, and marital status. Main outcome was ‘sitting time’ (collected by the Marshall questionnaire); chronic diseases and anthropometric measurements were registered.

**Results:**

464 patients, 58.4% women, mean age 51.9 years (SD 10.1), 76.1% married, 60% manual workers, and 48.7% had finished secondary education. Daily sitting time was 6.2 hours on working days (374 minutes/day, SD: 190), and about 6 hours on non-working ones (357 minutes/day, SD: 170). 50% of participants were sedentary ≥6 hours. The most frequent sedentary activities were: working/academic activities around 2 hours (128 minutes, SD: 183), followed by watching television, computer use, and commuting. Men sat longer than women (64 minutes more on working days and 54 minutes on non-working days), and individuals with office jobs (91 minutes),those with higher levels of education (42 minutes), and younger subjects (25 to 35 years) spent more time sitting.

**Conclusions:**

In our study performed in overweight/moderately obese patients the mean sitting time was around 6 hours which was mainly spent doing work/academic activities and watching television. Men, office workers, individuals with higher education, and younger subjects had longer sitting time. Our results may help design interventions targeted at these sedentary patients to decrease sitting time.

## Introduction

Obesity is a major public health problem that in 2008 already affected half a billion adults worldwide. Defined by the World Health Organization (WHO) as a body mass index (BMI) of ≥30 kg/m^2^ (a BMI of 25–34.9 kg/m^2^ is considered overweight) [1], it is one of the leading causes of mortality. According to the WHO, more than 2.8 million adults die each year because of obesity/overweight and worldwide percentages have significantly risen over the past years [2].

Overweight/obese individuals perform less physical activity and spend more time each day sitting [3;4]. Most daily life activities that are spent sitting are considered sedentary behavior. Sedentary behavior is defined as any waking behavior characterized by low energy expenditure (≤1.5 Metabolic Equivalent Units, METs) while in a sitting or reclining posture [5].It is becoming increasingly prevalent in our society and could even come to occupy more than 50% of adults’ waking time [6;7]. Among the pursuits carried out whilst sitting, television viewing, computer use (especially at work), and motorized journeys stand out [8–10].Such behavior has negative health consequences, and time spent sitting (>6 vs. <3 hours/day) is associated with mortality in both women and men [11]. In some studies such as that of Patel et al. [11] it has been independently associated with total mortality, regardless of physical activity level, however, the literature about this topic is inconclusive.

Both conditions, overweight/obesity and prolonged sitting time are associated with increased mortality [2;8;12] and chronic diseases such as diabetes mellitus type II, metabolic syndrome, cardiovascular disease, osteoporosis, and some cancers [11;13;14].Nevertheless, in many cases patients who are obese/overweight are unaware of their sitting time and its consequences [9].

Any increase in physical activity is potentially useful to reduce weight [15]. Current interventions are based on diet, exercise, and psychological support, however, they have limited long-term efficacy because of low adherence to moderate to vigorous physical activity programs [4;16]. Nevertheless, a reduction in prolonged sitting time can improve health, and reduce obesity consequences, irrespective of the level of the individual’s physical activity [17]. Another approach to help the overweight/obese become more active could be to encourage reduced sitting and increased light intensity physical activity levels [18]. In addition, identifying motivation to change sedentary behavior and the actual stages involved [19;20] may aid primary healthcare professionals design targeted interventions to reduce sedentary behavior for these patients.

To date, there is limited evidence regarding sedentary behavior in overweight/obese individuals in the Catalan population. Previous studies have assessed sitting time in general populations [21;22] with a wide variation amongst countries. The characteristics most related to longer sitting have been reported to be age and a higher level of education [6;22;23]. To the best of our knowledge, however, none of the studies has examined the profile of sedentary behavior in overweight/obese individuals and its association with health outcomes.

This descriptive study was conducted to ascertain sitting time, the profile of sedentary behavior in overweight and moderately obese adults attending primary care visits in Catalonia and their association with health, socio-demographics, occupation, and education level.

The study forms part of a clinical trial, Sedestactiv, the protocol of which has been previously published [24]. It aims to assess the effectiveness of a primary healthcare education-based intervention among the overweight and moderately obese in terms of reducing sitting time.

Prior to designing the clinical trial intervention it was necessary to ascertain sitting time, the sedentary profile of this population, and their association with some key aspects: health, socio-demographic status, occupation, and level of education. For this reason, an observational study was designed. Moreover, in order to understand better the motivation required to decrease this population’s sitting time a qualitative study was performed to identify both barriers and enabling factors to decrease such behavior [9].

## Materials and methods

### Study design

A descriptive, multicenter study was performed in 25 primary healthcare centers (PHC) from different regions of Catalonia (Spain) between July and December 2012. One hundred and thirty health professionals voluntarily took part. All the researchers were physicians and nurses from the PHCs which helped participation in the study given the healthcare professionals’ proximity and knowledge of the patients. All the researchers received an email inviting them to join the study and they were sent a procedure manual with information on how to select the participants.

Inclusion criteria of participants included: (a) men and women aged 25–65 years who attended at the PHC for any reason; and (b) a diagnosis of overweight or moderate obesity (BMI: 25–34.9 kg/m2). Exclusion criteria included: certain medical conditions which could contraindicate physical activity, patients who did not speak Catalan or Spanish, and those residing outside the study area.

### Ethics statement

The study protocol was reviewed and approved by the Health Care Ethics Committee and the Clinical Research Ethics Committee of the Primary Health Care University Research Institute-IDIAP Jordi Gol located in Barcelona, Spain. Written informed consent was obtained from all patients prior to participation.

### Sample size calculation

Sample size was calculated according to the aim of the project: to assess the prevalence of sitting time in the overweight/moderately obese. Accepting a confidence interval of 0.95 for an accuracy of +/- 0.05 units (p = q = 0.5), a population-based random sample minimum of 452 subjects was required. A 15% restock (calculated according Granmo Online program,http://www.imim.es/ofertadeserveis/software-public/granmo/) was estimated to obtain this sample size. The final sample included 464 participants.

### Outcome measures

The following information was obtained by healthcare professionals through face-to-face interviews: age, sex, educational level, occupational social class, and civil status. The main outcome (sitting time) was collected by the Marshall specific questionnaire [25]. This is a tool that assesses time spent sitting (hours and minutes) on weekdays and weekends in the following domains: (a) while traveling to and from places (e.g., work, shops); (b) while at work; (c) while watching television; (d) while using a computer at home; and (e) at leisure, not including watching television (e.g., visiting friends, movies, eating out). Sitting time was considered prolonged if it was 6 hours or more a day.

To assign occupational social class we used the Spanish classification based on Goldthorpe’s scheme which was designed to facilitate international comparisons [26]. It includes five well-established main social groups which were subsequently summarized into two categories: manual workers (social classes III M, IV-V) and workers with office jobs (the rest of the categories) for analysis [26]. Social class was assigned through the current or prior occupation of the patient; in cases where the subject had not worked, through the current or prior occupation of the head of the household [27].

Information was collected from medical records on relevant chronic diseases (hypertension, dyslipidemia, endocrine diseases such as type 2 diabetes, vascular, cardiological, lung, bone and joint diseases, cancer, depression, and fibromyalgia) which could influence sedentary behavior. Tobacco consumption, and the intention of the participants to change their sedentary behavior, were also registered and codified according to the categories of Prochaska and Di Clemente, based on a closed question [18]. Finally, weight, height, and BMI were recorded.

Variables were gathered by an electronic questionnaire on the “Surveymonkey” platform which ensured confidentiality of data. The electronic questionnaire recorded the randomization process and the methods employed for each variable.

The characteristics of participants are presented in the results section.

### Statistical analysis

A description of all the analyzed outcomes was performed, summarizing the qualitative variables by frequency (n) and percentage (%), and quantitative variables by mean and standard deviation (± SD). For the main outcome ‘sitting time’, distribution by median and percentiles was also analyzed. The relationship between sitting time and the rest of the variables was analyzed by comparing the means, the T-student test was employed for two groups and ANOVA for more than two.

Multiple linear regression models were performed to evaluate the joint effect of all statistically significant variables on sitting time. Forward conditional was employed to select the variables for the models which were validated by checking the normality of residuals with the Kolmogorov-Smirnov test. Values <0.05 p were considered statistically significant. The analysis was performed with SPSS Statistics for Windows, Version 17.0. Chicago: SPSS Inc.

## Results

### Participant characteristics

The study included 464 participants (58.4% women) with a mean age of 51.9 years (SD: 10.1) (Table 1). Participants were more likely to be married (76.1%), manual workers (60.34%), and 48.7% had completed, at least, secondary education. The most prevalent chronic diseases were hypertension (44.4%) and dyslipidemia (41.2%), and 22.84% of the patients were disabled. 47.6% of the subjects were not planning to decrease their sitting time (pre-contemplation phase).

**Table 1:** Characteristics of the analyzed population.

Variable	N	%
**Gender**		
Men	193	41.6%
Women	271	58.4%
Age Group		
25 to 35	43	9.3%
36 to 45	71	15.3%
46 to 55	138	29.7%
56 to 65	212	45.7%
Mean age ±SD	51.9±10.1	
**Employment status**		
Student	4	0.9%
Housewife	84	18.1%
Employed	254	54.7%
Unemployed	47	10.1%
Permanent disability/ incapacity	28	6.0%
Retired	38	8.2%
Don’t know/No response	9	1.9%
**Educational level**		
Primary studies or less	238	51.3%
Secondary studies or higher	226	48.7%
**Civil status**		
Single/widow(er)/separated	111	23.9%
Married	353	76.1%
**Work category (current or previous employment)**		
I. University graduate or higher, large company director	23	4.96%
II. Qualified worker, small company director, manager, farmer	38	8.19%
III M. Skilled blue-collar worker (e.g. carpenter, electrician)	70	15.09%
III NM. Skilled white-collar worker (e.g. secretary, shop assistant)	107	23.06%
IV. Semi-skilled worker (e.g. postman, bus driver)	60	12.93%
V.- Unskilled worker (e.g. road sweeper, cleaner, doorman)	150	32.33%
VI. Don’t know/ no answer	16	3.44%
**Work category (Manual/office worker)**		
Office (I,II,III NM)	168	36.21%
Manual (III M,IV,V)	280	60.34%
**Concomitant pathologies**		
Hypertension	206	44.4%
Dyslipidaemia	191	41.2%
Endocrine	85	18.3%
Osteo-articular	67	14.4%
Depression	51	11.0%
Fibromyalgia	24	5.2%
Vascular	18	3.9%
Cardiological	17	3.7%
Pulmonary	17	3.7%
Cancer	5	1.1%
**Type of pathology (Disabling/non-disabling)**		
Disabling (osteo-articular, fibromyalgia, vascular, pulmonary, Or cancer)	106	22.84%
None or non-disabling	358	77.16%
**Willingness to reduce sitting time**		
Pre-contemplation	221	47.6%
Contemplation	85	18.3%
Preparation	45	9.7%
Action	61	13.1%
Maintenance	49	10.6%
Relapse	3	0.6%

Table 2 shows the descriptive sample of the population analyzed in relation to sitting time, more or less than 6 hours on working and non-working days.

**Table 2:** Characteristics of the analyzed population with respect to sitting time, more or less than six hours, on working and non-working days.

	Working days		Non-working days	
	N	%	N	%
**Sitting time**				
Less than 6 hours: n (%)	234	50.4	233	50.2
More than or the same as 6 hours: n (%)	230	49.6	231	49.8

Table 3 presents the descriptive sample of the daily sitting time on working and non-working days. On weekdays the average sitting time was 374 minutes (SD: 190), representing 6.2 hours. The single activity for which more sitting time was reported was that dedicated to work and/or academic activities which represented an average of around 2 hours (128 minutes/day, SD: 183),34% of total sitting time. This was followed by time spent watching television, using the computer, and finally, transport. On non-working days, the average sitting time was 357 minutes/day (SD: 170), about 6 hours. It is noteworthy that the activity which was reported to take up the most sitting time was watching television with a mean of 3 hours (178 minutes/day, SD: 98), which represented 50% of total sitting hours.

**Table 3:** Global sitting time (minutes) and main sedentary activities on working and non-working days.

Quantitative variable	Mean	SD	Q1*	Median	Q3**	Hours	(%)
**Sitting time in minutes/day**							
**Working days**	373.9	190.8	238.3	352.5	493.0	6.2	
By sedentary activity							
Transport	44.5	61.9	0.0	30.0	60.0	0.7	(11.9)
Work or academic activities	128.1	183.9	0.0	0.0	240.0	2.1	(34.3)
Computer use at home	65.7	119.5	0.0	0.0	60.0	1.1	(17.6)
Watching television	125.3	78.0	60.0	120.0	180.0	2.1	(33.5)
Rest of the time	53.8	62.0	0.0	35.0	90.0	0.9	(14.4)
**Non-working days**	357.4	170.4	240.0	358.5	467.5	6.0	
By sedentary activity							
Transport	34.3	45.5	0.0	15.0	60.0	0.6	(9.6)
Work or academic activities	39.3	89.1	0.0	0.0	0.0	0.7	(11.0)
Computer use at home	47.3	79.1	0.0	0.0	60.0	0.8	(13.2)
Watching television	178.4	98.2	120.0	180.0	240.0	3.0	(49.9)
Rest of the time	72.5	80.2	0.0	60.0	120.0	1.2	(20.3)

*Q1: First quartile

**Q3:Third quartile

Table 4 describes a comparison of the sitting time means with respect to the characteristics of the analyzed population. It was observed that sitting time varied with statistical significance (p-value <0.001) between genders, with more sitting time in men on both working, 414 minutes/day (SD: 190), and non-working days, 389 minutes/day (SD: 177). Declared sitting time decreased with age (p-value <0.001), especially on working days. In addition, depending on the type of occupation, it was greater in the employed (413 minutes/day, SD: 210) than housewives (302 minutes/day, SD:144) on working days (p-value <0.001). With respect to type of employment, office workers reported 457 minutes/day (SD: 195) sitting time on working days, manual workers were seated 329 minutes/day (SD: 174), with p-value <0.001.

**Table 4:** Comparison of mean sitting time (minutes) with respect to the characteristics of the analyzed population.

		Working day			Non-working day		
Variable	N	Mean	±SD	p value	Mean	±SD	p value
Total	464	373.9	191.0		357.4	170.4	
**Gender**							
Men	193	414.5	189.7	<0.001	389.4	177.2	<0.001
Women	271	345.0	186.6		334.7	161.9	
**Age group**							
25 to 35	43	465.1	226.2	<0.001	388.6	194.5	<0.001
36 to 45	71	409.3	192.0		377.6	192.2	
46 to 55	138	384.3	206.1		365.3	161.9	
56 to 65	212	336.7	162.2		339.3	161.9	
**BMI**							
Overweight (BMI: 25–30)	275	368.4	187.8	0.456	353.7	169.7	0.568
Obesity (BMI>30)	189	381.9	195.3		362.9	171.8	
**Employment status**							
Housewife	84	302.0	144.4	<0.001	340.7	152.3	0.59
Employed	254	413.5	210.3		359.8	181.8	
Unemployed and permanent disability/incapacity, retired and don’t know/no response	126	342.0	154.2		363.8	158.3	
**Educational level**							
Primary studies or less	238	315.5	165.0	<0.001	328.4	149.2	<0.001
Secondary studies or higher	226	435.4	197.1		388.0	185.7	
**Civil status**							
Single/ widower/ divorced	111	393.7	212.1	0.1930	365.3	190.0	0.56
Married	352	366.7	182.9		354.6	164.1	
**Work category (current or previous employment)**							
I. University graduate or higher, large company director	23	579.3	185.5	<0.001	431.1	196.2	0.04
II. Qualified worker, small company director, manager, farmer	38	488.5	150.2		440.4	203.6	
III M. Skilled blue-collar worker (e.g. carpenter, electrician)	70	341.6	178.5		373.5	192.2	
III NM. Skilled white-collar worker (e.g. secretary, shop assistant)	107	420.8	199.5		349.8	160.2	
IV. Semi-skilled worker (e.g. postman, bus driver)	60	398.9	193.5		351.5	157.9	
V. Un-skilled worker (e.g. road sweeper, cleaner, doorman)	150	295.8	155.0		329.1	153.9	
VI. Don’t know/ no answer	16	272.1	104.4		323.4	128.7	
**Work category (Manual / office worker)**							
Office (III, IV, V)	168	457.8	194.7	<0.001	381.4	179.7	0.065
Manual (I, II, III)	280	329.3	174.1		345.0	165.5	
**Concomitant pathologies**				** **			
Hypertension	206	365.3	179.2	0.39	362.5	176.5	0.57
Dyslipidaemia	191	367.4	182.2	0.54	369.8	163.9	0.19
Endocrine	85	340.1	172.2	0.71	355.5	189.2	0.90
Osteo-articular	67	349.9	155.5	0.26	357.2	181.4	0.99
Depression	51	347.6	169.2	0.29	350.5	155.8	0.75
Fibromyalgia	24	324.9	176.5	0.19	340.1	164.9	0.60
Vascular	18	280.4	147.7	0.34	365.1	165.3	0.84
Cardiological	17	370.5	136.1	0.9	389.0	136.1	0.44
Pulmonary	17	342.6	214.4	0.49	314.1	176.1	0.28
Cancer	5	343.6	155.2	0.72	329.6	149.9	0.71
**Disabling pathology (yes/no)**							
Yes (Osteo-articular, fibromyalgia, vascular, pulmonary, cancer)	106	328.7	153.5	0.005	345.0	166.0	0.395
No	358	387.3	198.7		361.1	171.8	
**Willingness to reduce sitting time**							
Pre-contemplation	221	337.0	179.7	0.010	341.0	165.3	0.17
Contemplation	85	432.5	209.0		390.4	178.6	
Preparation	45	417.5	184.8		376.3	202.6	
Action	61	385.3	183.0		360.7	164.8	
Maintenance	49	375.8	182.8		345.2	150.3	
Relapse	3	515.0	330.9		485.0	70.9	
**Smoking status**				** **			
Smoker	95	398.3	183.9	0.027	389.8	181.7	0.116
Ex-smoker	125	378.5	183.0		348.1	165.5	
Non-smoker	244	362.0	197.0		379.6	167.5	

There was a statistically significant difference (p = 0.010) between individuals who planned to reduce their sitting time (contemplative phase) and those who did not (pre-contemplation phase). Individuals who were in the contemplative phase sat longer on working days (95 minutes/day more than individuals in pre-contemplation phase). Non-smokers sat less than smokers/former smokers on working days (p-value = 0.027).

**Fig 1:**
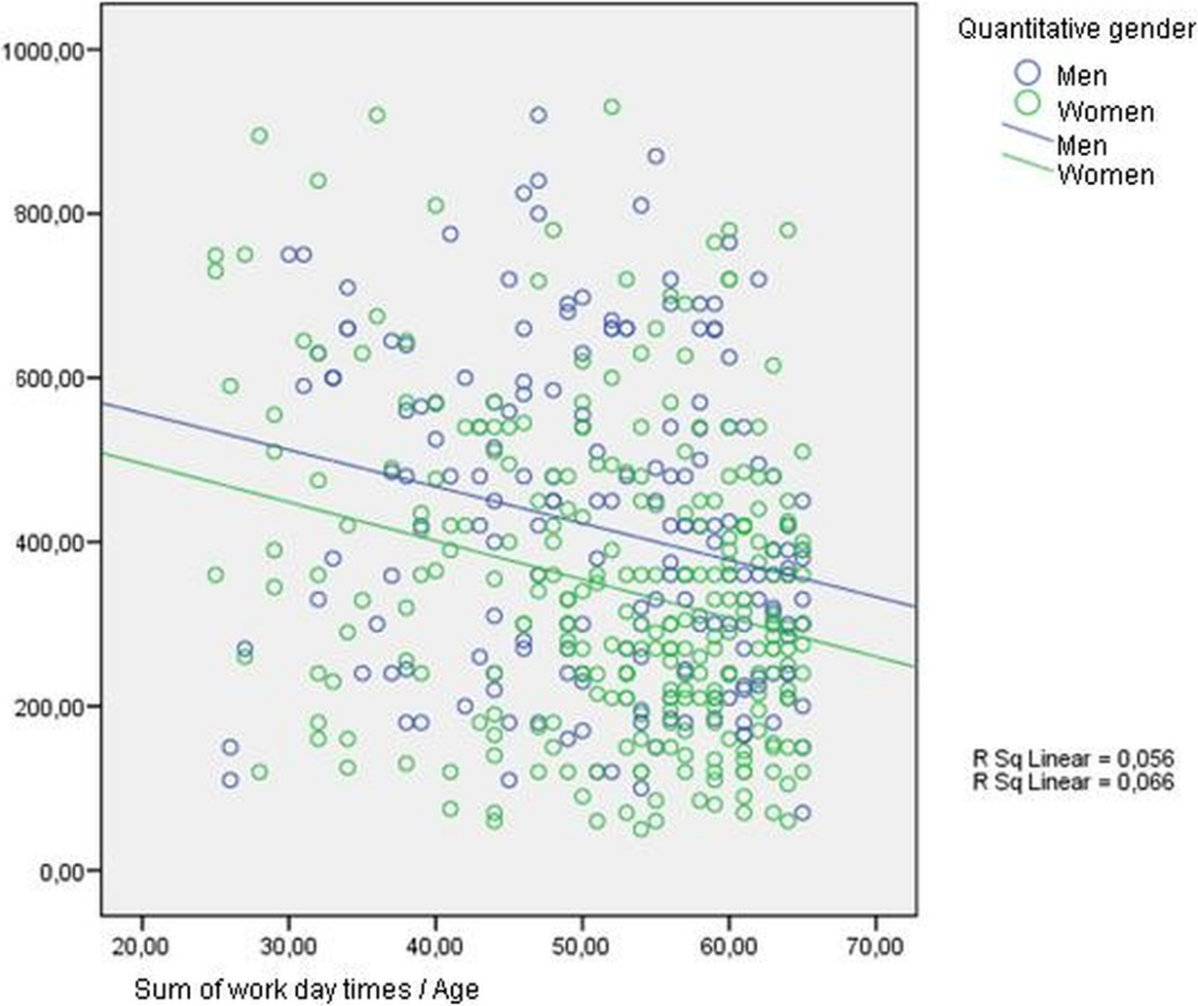
Linear regression coefficients of sitting time in work day by gender.

Table 5, linear regression coefficients of the predictors of sitting time, shows adjusted linear models of the variables that attained statistical significance regarding sitting time. Men sat longer in general:64 minutes more on working days (95% CI 31.9–96.1) and 54 minutes more on non-working days (95% CI 24–85). On working days longer sitting time was related to: being currently employed 44minutes more (95% CI 10.5–78.3) compared to housewives and students; working in an office91 minutes more (95% CI 53.7–129.7) compared to manual jobs; and secondary/higher studies 42 minutes more(95% CI 4.2–81.1). In addition, a younger age was associated with greater sitting time (in adults it decreases by 2 minutes/year) (See on Fig 1).

**Table 5:** Linear regression coefficients of sitting time predictors (in minutes).

	Variables	Coefficient	p value	95% Confidence Interval	
				(Lower limit -	(Upper limit)
**Working days(1)**					
	(Constant)	383.5		(279.6-	487.4)
	Office work (Reference: Manual)	91.7	<0.001	(53.7-	129.7)
	Gender: Male	64.0	<0.001	(31.9-	96.1)
	Age (years)	-2.2	0.013	(3.9-	-0.5)
	Employment (Reference: Housewife / student)	44.4	0.010	(10.5-	78.3)
	Educational level: Secondary or higher	42.6	0.030	(4.2-	81.1)
**Non-working days (2)**				
	(Constant)	310.5	<0.001	(286.5-	334.5)
	Gender: Male	54.6	<0.001	(24.0-	85.1)
	Educational level: Secondary or higher	48.9	0.002	(18.0-	79.9)
R Square model 1: 0.443 / R square model 2: 0.225.					

## Discussion

In our study, which was carried out in overweight/moderately obese individuals aged 25 to 65 years, nearly half the population (49.6%) sat ≥6 hours a day. This percentage is higher than in the general population as demonstrated in a 2013 European survey [26] where 37% of the population was sitting ≥5.30 hours (26% between 5.30 and 8.30 hours, and 11% ≥8.30 hours).

The average sitting time in our study was found to be around 6.2 hours on working days (374 minutes/day, SD: 190) and about 6 hours on non-working ones (357 minutes/day, SD: 170). In this overweight/ moderately obese population, the sitting time was rather higher than in other general population studies which reported between 5 and 6 hours/day. For instance, Bauman et al. [21] performed a study in 20 countries with 50,000 individuals aged 18 to 65 years. They observed that the average sitting time was 346 minutes/day, with a wide variation among countries. In addition, Bennie et al. [22], who assessed a general European population aged 15 to 98 years, reported an average of 309 minutes/day (SD: 185). Differences were found with respect to geographic pattern: there was a greater proportion of sitting time in northwestern European countries, as such as the Netherlands and Denmark (376–407 minutes/day), and a smaller proportion in Southern European countries, such as Portugal and Malta (191–236 minutes/day). Regarding Spain, the study [22] reported a global sitting time of 284 minutes/day (95% CI 274–294). Variations with respect to our findings may be due to differing target populations: our study only included overweight/moderately obese adults aged 15 to 65 years. In addition, the previous studies had been carried out some time ago, the study by Bennie et al was performed with data collected in 2005, and it is probable that daily sitting time has increased in recent years.

The activities on working days in which the participants spent the most time sitting were: working/ academic activities for about 2 hours, followed by watching television, use of the computer, and transport. On non-working days, watching television was the most common sedentary activity a fact that is consistent with statistics compiled for adults in the United Kingdom [28], the United States [29], and Australia [30] which demonstrate that it is the most prevalent leisure activity.

According to our study, individuals watched television on working days for 128 minutes and on non- working ones for 178 minutes, the latter representing 50% of the total average sitting time. Such figures concur with other studies in European general populations, for instance in Belgium in 2010where the average time watching television was 128.40 minutes/day (SD: 76.74) [31], in the United Kingdom with 157 minutes/day [28], and France 2 to 3 hours a day [32]. A North American population, however, was reported to watch television for a longer time, with an average between 4 hours and 5 hours according to a 2009 study concerning the use of television, internet, and mobile phones [33]. A progressive increase of television watching time with respect to age, and an increment from the previous year, were also observed [29].

Television watching is the sedentary behavior that has been most researched [34]. The factors that have been associated with increased viewing time are obesity [33–35], lower level of education [33;34;36], being older, unemployment, and working fewer hours. Moreover, a poor environmental infrastructure, such as few pedestrian areas, and neighborhoods unsuitable for walking (poorly communicated streets, large parking areas) have been observed to increase the time women spend watching television [37].

Regarding socio-demographic, laboral, and educational factors, the individuals who spent the most time seated on a daily basis in our study were the youngest participants (aged 25 to 35 years), male, office workers, and individuals with a higher level of education. These overall findings are consistent with other authors although with variations in the age of the group that spend more time seated [22;23;38].

We observed that with respect to gender, sitting time was statistically significantly greater in men on working (64 minutes more) and non-working days (54 minutes).Other studies are in agreement with our results, Patel et al. [11] demonstrated that men spent more time seated, especially in the group >6 hours per day, which corresponded to the category with the greatest health risk. We also found studies with contrasting results, such as Matthews et al. [6] who reported that women spent more time seated than men although the pattern changed when they were older than 60 years.

In relation to age, young people between 25 and 35 years were those who spent more time seated. With the increase of age declared sitting time decreased progressively (2 minutes/year). It should be noted that in our study, participants were between 25 and 65 years, so results cannot be extrapolated to other groups of different ages. According to Bennie et al. [22], whose study population was between 15 and 98 years old, it was the young people (between 18 and 24 years) who spent more time seated. Matthews et al. [6] found that the groups which spent more time seated were older teenagers and adults >60 years. In particular, the group between 70 and 85 years was the most sedentary of all: > 9 hours/day. Authors such as Harrington et al. [23] and Bauman et al. [21] also showed that sitting time augmented with age.

Regarding employment, it was observed that workers spent more time seated (44 minutes more than students and housewives), and especially those with an office job (91 minutes longer than those who perform manual jobs).

In addition, individuals with a higher level of education (secondary or tertiary education) spent more time seated (42 minutes more than those with less education). These results are similar to other studies such as Bauman et al. [21], Chau et al. [39], and Harrington et al. [23]. Most professions requiring a higher level of education are sedentary, while manual work is usually performed by individuals with less education.

With respect to weight, as already mentioned, many studies have demonstrated that the overweight and obese sit longer than individuals with normal weight [40;41] during both leisure time/weekend [42] and in daily life, especially watching television [33;35;37].We found no differences between overweight and obese participants. Although our results showed that the moderately obese spent more time seated than the overweight, these differences were not statistically significant (p = 0.45). In the United States, Harrington et al. [23] reported, with statistically significant differences, that obese women spent more time seated that those who were over-or normal weight (311, 261, and 263 minutes/day, respectively).

In relation to health status in our study, individuals with a disabling pathology were not found to spend more time sitting. This is in contrast to Bennie et al. [22] who observed that adults reporting worse health status (poor or very poor) were those who stayed sitting longer. Our findings may be due to the fact that participants with contraindications for physical activity were excluded from the study.

Regarding willingness to reduce sitting time, 47.6% of the participants had not taken this behavioral change into consideration (pre-contemplation phase). According to Van Dick et al.[36], factors that can help reduce sitting time include self-confidence in being able to limit the time spent watching television/using the computer, and being aware not only of the harm of such behavior but also the benefits of changing it.

### Limitations of the study

Certain limitations are inherent in the study design:

1) This is a cross-sectional study which, whilst allowing us to observe the most prevalent characteristics of the participants seated for the longest time and describe associations, does not permit a cause-effect relationship to be established.
2) The sample selected (aged between 25 and 65 years, overweight/moderately obese, and receiving primary health care) does not allow us to extrapolate our results to other populations(different obesity levels/normal weight). Nonetheless, the sample proved to be very useful for the design of SEDESTACTIV clinical trial (SEDESTACTIV, NCT01729936) and other interventions aimed at reducing the amount of sitting time for this profile of primary healthcare patients [24].

Participants aged 25 to 65 years were selected because we wished to include adults of working age with the possibility of accepting preventive changes in their sedentary behavior. Individuals aged less than 25 years were excluded as this age range goes very infrequently to primary healthcare consultations. Neither were those aged over 65 years included as they tend to present chronic pathologies, especially osteoarticular diseases, which hinder the possibility of maintaining less sedentary behavior.

3) Sitting time was evaluated on the basis of a validated questionnaire (Marshall) [25]. Data would, however, have been more accurate employing an objective measurement such as an accelerometer or inclinometer.When compared with objective instruments, self-referral measures may underestimate sitting time [17;43]. As for the type of tool, inclinometers evaluate the position and capture more accurately sitting time in comparison with accelerometers.
4) In the Discussion the total sitting time obtained in our study was compared with other authors who had employed different questionnaires. The sum of several domains tends to be higher than from one question (e.g. IPAQ). It would, therefore, have been more suitable to compare our results with others who had also used the Marshall questionnaire [25].However, to our best knowledge, this questionnaire has not been used to describe the profile of sedentary behavior in overweight/obese individuals and its association with health or, socio-demographics outcomes.

## Conclusions

In summary, our findings indicate that: (a)nearly half the overweight/obese spend ≥ 6h/day seated; (b) men who have office jobs and higher levels of education, and younger adults, spend more time seated; (c) activities involving more sedentary time include employment/academic activities on working days, and watching television on non-working days.
