# Correction: Patterns of sedentary behavior in overweight and moderately obese users of the Catalan primary-health care system

**DOI:** 10.1371/journal.pone.0195312

**Published:** 2018-03-28

**Authors:** Elena Martínez-Ramos, Angela-Maria Beltran, Carme Martín-Borràs, Lourdes Lasaosa-Medina, Jordi Real, José-Manuel Trujillo, Mercè Solà-Gonfaus, Elisa Puigdomenech, Eva Castillo-Ramos, Anna Puig-Ribera, Maria Giné-Garriga, Noemi Serra-Paya, Beatriz Rodriguez-Roca, Ana Gascón-Catalán, Carlos Martín-Cantera, for the SEDESTACTIV group

There are errors in the Funding section. The correct funding information is as follows: The study was supported by research grants from Carlos III Institute of Health, Ministry of Economy and Competitiveness (Spain), within the Technical, Scientific and Innovation Research National Plan, with reference PI11/01082, RD16/0007/0001, co-funded with European Union ERDF funds (European Regional Development Fund)”, VI Catedra of the European University of Madrid, Funding for the realization of doctoral theses “Isabel Fernández” 2015 SEMFYC, Ajut XB 2012 Unitat de Suport a la Recerca de Barcelona de l’Institut Català de la Salut and 14th Financial aid for training in research and doctorate in primary care IDIAP Jordi Gol 2013. The funders had no role in study design, data collection and analysis, decision to publish, or preparation of the manuscript.
